# Hot spot or not: a comparison of spatial statistical methods to predict prospective malaria infections

**DOI:** 10.1186/1475-2875-13-53

**Published:** 2014-02-11

**Authors:** Jacklin F Mosha, Hugh JW Sturrock, Brian Greenwood, Colin J Sutherland, Nahla B Gadalla, Sharan Atwal, Simon Hemelaar, Joelle M Brown, Chris Drakeley, Gibson Kibiki, Teun Bousema, Daniel Chandramohan, Roland D Gosling

**Affiliations:** 1 National Institute for Medical Research (NIMR), Mwanza Medical Research Centre, Mwanza, Tanzania; 2 The Global Health Group, University of California, San Francisco, CA, USA; 3 Faculty of Infectious and Tropical Diseases, London School of Hygiene and Tropical Medicine, London, UK; 4 Department of Medical Microbiology, Radboud University Nijmegen Medical Centre, Nijmegen, The Netherlands; 5 Department of Epidemiology and Biostatistics, University of California San Francisco, San Francisco, CA, USA; 6 Kilimanjaro Clinical Research Institute and Kilimanjaro Christian Medical College, Kilimanjaro, Moshi, Tanzania

**Keywords:** Spatial methods, Malaria, Transmission, Hotspots, Micro-epidemiology, Serology, PCR, Africa, *Plasmodium falciparum*

## Abstract

**Background:**

Within affected communities, *Plasmodium falciparum* infections may be skewed in distribution such that single or small clusters of households consistently harbour a disproportionate number of infected individuals throughout the year. Identifying these hotspots of malaria transmission would permit targeting of interventions and a more rapid reduction in malaria burden across the whole community. This study set out to compare different statistical methods of hotspot detection (SaTScan, kernel smoothing, weighted local prevalence) using different indicators (PCR positivity, AMA-1 and MSP-1 antibodies) for prediction of infection the following year.

**Methods:**

Two full surveys of four villages in Mwanza, Tanzania were completed over consecutive years, 2010-2011. In both surveys, infection was assessed using nested polymerase chain reaction (nPCR). In addition in 2010, serologic markers (AMA-1 and MSP-1_19_ antibodies) of exposure were assessed. Baseline clustering of infection and serological markers were assessed using three geospatial methods: spatial scan statistics, kernel analysis and weighted local prevalence analysis. Methods were compared in their ability to predict infection in the second year of the study using random effects logistic regression models, and comparisons of the area under the receiver operating curve (AUC) for each model. Sensitivity analysis was conducted to explore the effect of varying radius size for the kernel and weighted local prevalence methods and maximum population size for the spatial scan statistic.

**Results:**

Guided by AUC values, the kernel method and spatial scan statistics appeared to be more predictive of infection in the following year. Hotspots of PCR-detected infection and seropositivity to AMA-1 were predictive of subsequent infection. For the kernel method, a 1 km window was optimal. Similarly, allowing hotspots to contain up to 50% of the population was a better predictor of infection in the second year using spatial scan statistics than smaller maximum population sizes.

**Conclusions:**

Clusters of AMA-1 seroprevalence or parasite prevalence that are predictive of infection a year later can be identified using geospatial models. Kernel smoothing using a 1 km window and spatial scan statistics both provided accurate prediction of future infection.

## Background

Malaria transmission in endemic countries is heterogeneous over multiple spatial scales [1,2]. At the micro scale, *P. falciparum* infections are frequently clustered in relatively few households that consistently have significantly more infections than others [3,4]. Many factors can contribute to this increased risk of malaria exposure, including design of housing, the proximity to mosquito breeding sites, host genetic factors, poor access to treatment, maternal education, wealth, and other as yet undefined characteristics [3,5-8]. At sites with very low levels of transmission, such as those found in Swaziland, cases of symptomatic malaria detected at health facilities can help in identification of a hotspot, as additional asymptomatic cases can be found living in close proximity to the index case [9]. In areas of moderate transmission intensity, malaria hotspots may provide a reservoir of infected human hosts that can maintain some transmission year round. The individuals in such hotspots are thus likely to have acquired anti-parasite immunity and to carry parasites without clinical symptoms. In the wet season, when the mosquito population increases, these clusters of asymptomatic carriers may be responsible for seeding transmission to the rest of the community, including less immune people who are more likely to suffer symptomatic infections [7]. Thus in these settings, hotspots are difficult to identify using the distribution of clinical (symptomatic) malaria cases alone.

The most used geospatial method to detect clusters of infection is the spatial scan statistic [10-12]. Measures of exposure which have been explored using spatial scan statistics include prevalence of infection, incidence of clinical malaria and serological markers of malaria exposure [13-18]. While this approach allows identification of clusters using statistical hypothesis testing, it may ignore more subtle small-scale spatial heterogeneity and clusters that do not fit within circular or elliptical windows [19]. An alternative method that has been used to detect clustering of infection is distance-weighted prevalence of infection, whereby infection prevalence in neighbours is used as a proxy measure for household level exposure [20,21]. This method allows for a smoother estimation of risk in space than spatial scan statistics.

This study seeks to determine which geospatial method best describes a malaria transmission hotspot by comparing methodologies using cross-sectional data collected during the first year of the study to predict the distribution of infections found in the second year.

## Methods

### Study site

**Figure 1:**
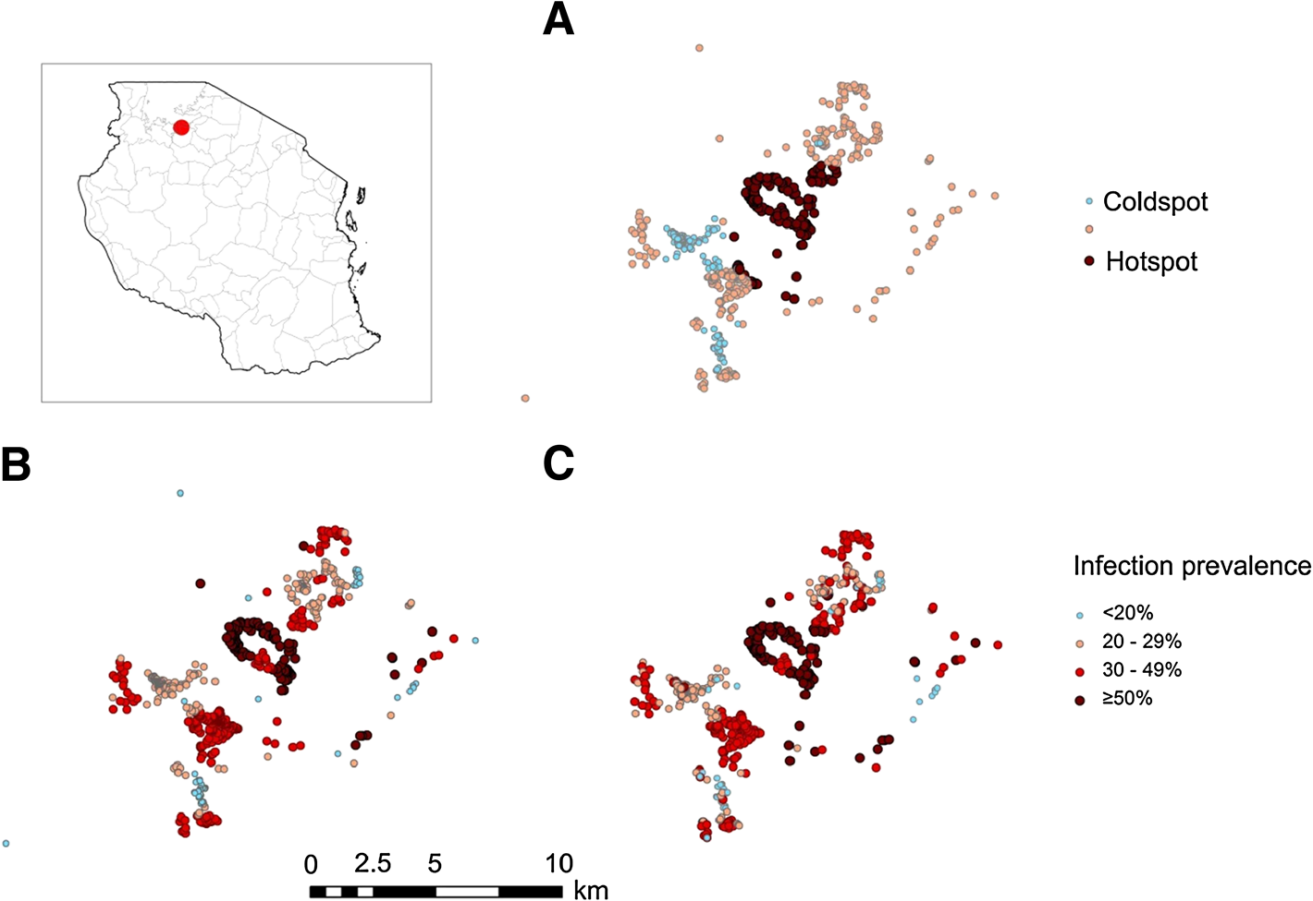
**Location of study site within Tanzania (inset map) and clustering of malaria infection using different methods. (A)** derived from SaTScan (coldspot significantly lower infection, hotspot significantly greater infection), **(B)** derived from Kernel and **(C)** derived from Weighted Local Prevalence.

Misungwi district (lat 2.85000 S, long 33.08333 E) is located 60 km from Mwanza town in the north-west of Tanzania at an altitude of 1,178 m above sea level (see Figure 1). The district is rural with moderately intense malaria transmission; the overall prevalence of infection in the region is estimated to be 31.4% by microscopy in children 6 -59 months (Tanzania HIV and Malaria Indicator Survey 2008). The district has two annual rainy seasons, the long rains between February and May, and the short rains between November and December. The dry and relatively hot season falls between June and September. Malaria incidence peaks one to two months after the rains start. The National Malaria Control Programme (NMCP) carried out indoor residual spraying (IRS) in the study area during the period from late November 2010 to late January 2011.

### Data collection

A census of four villages in a single ward was carried out in the dry season, between August and early November 2010. All data were collected using personalized digital assistants and every household was visited and mapped using a global positioning system (GPS). All individuals in the ward were invited to participate in the study. The head of household gave information on the age, sex and insecticide-treated net (ITN) use of those who were not present. Individuals who consented to join the study were asked to provide a finger-prick sample of blood which was spotted onto Whatman® standard 3 mm filter paper for parasite detection and serological analysis. Subjects who reported having had fever within the previous 24 hours were tested for malaria using a histidine-rich protein 2 (HRP2) rapid malaria diagnostic test (RDT, *Paracheck*-*Pf*®, Orchid Biomedical Systems, Goa, India) and referred to a study clinician for management of their febrile illness.

A follow-up survey was carried out in the same study villages during August to November 2011, one year after the initial study. The same procedures were carried out during the second survey as during the baseline survey.

### Molecular estimation of *P. falciparum* infection

DNA was extracted from filter papers using the Chelex® (Sigma, USA) extraction method described previously [22] in 96 deep-well plates. Parasite DNA was detected using nested PCR (nPCR) targeting the 18S rRNA gene as previously described [23].

### Serology

Antibodies were eluted from filter paper spots and assayed for specific IgG responses to P.falciparum AMA-1 and MSP-1_19_ by ELISA as described by Corran *et al*. [24]. Samples were tested in duplicate. Duplicate optical density (OD) values OD values that differed by more than 1.5-fold were rejected and, if possible, rerun. For each plate a standard curve was generated from a known positive control and blank wells were included and OD values normalised to these. To define seroprevalence a mixture model was applied to the OD data which assumed two inherent Gaussian distributions; a narrow distribution or sero-negatives and a broader distribution of seropositives. A cut-off was calculated as the mean plus 3 standard deviations of the narrow distribution and was calculated separately for each antigen [25].

### Cluster analysis

While there are a range of different methodological approaches to identifying clusters of infection [12,26], here we focus on three geospatial cluster detection methods to explore baseline clustering of infection and serological markers and their ability to predict infection in the second year of the study. The unit of analysis was the individual, meaning that clustering of infected individuals was assessed rather than clustering of households with infection. Infection in the second year was defined as a positive nPCR result recorded as a binary variable.

### Satscan analysis

Spatial analysis was performed to assess possible clustering of nPCR-positive individuals. A spatial scan statistic was obtained using the Bernoulli model [11] and SaTScan software (SaTScan, version 8.2.1). This software applies multiple circular windows, which are plastic in both position and size, across the study area. Each distinct circle represents a possible cluster. For each circle, the number of observed and expected infected individuals are counted, with expected numbers calculated assuming an even distribution of infections across the population. As multiple infected and non-infected individuals can be specified at each household, the spatial distribution of households is accounted for. A likelihood ratio test is used to compare the prevalence of infection within the circle to that outside it to identify significant clusters of higher than expected (hotspot) or lower than expected (coldspot) prevalence. The statistical significance of this hotspot is evaluated taking into account the multiple tests for the many potential cluster locations and sizes evaluated as well as the distribution of the population [10]. The maximum proportion of the population that a cluster could contain was set at 50%. This method has been extensively explored in studies of the micro-epidemiology of malaria [12,13,27-29].

Households were grouped into three categories: 1) hotspots (clusters of significantly higher than expected malaria prevalence); 2) coldspots (clusters of significantly lower than expected malaria prevalence); and, 3) all other households. Clusters were defined using three measures: 1) nPCR positivity; 2) antibody seropositivity to AMA-1; 3) antibody sero-positivity to MSP-1_19_; and, 4) antibody seropositivity to AMA-1and/or MSP-1_19_. So as to make results from analyses using different clustering methods comparable, hotspots were assigned a score of 1, coldspots 0 and all remaining households a score of 0.5. Households for which data were only available in the second year were assigned a hotspot score according to whether the household lay within the radius of the hot or coldspot.

### Kernel analysis

Kernel density estimation is a statistical procedure used to produce a smoothed estimate of density of events, such as individuals, across space [26]. For any given point, the density of events within a predefined window is estimated, with the influence of events weighted according to the distance from the centre of the window. The weight assigned to each event is derived from the kernel function applied. In this analysis a quadratic kernel function was used with an initial window radius of 1 km. A quadratic function allows importance of data from neighbouring households to be relative to the distance to the index household. To obtain a smoothed estimate of infection prevalence over the study region, a kernel density surface of numbers nPCR positive was divided by a kernel density surface of numbers examined. This resulted in each household having a value between 0 (least exposed households) and 1 (most exposed households). Households for which data were only available in the second year were assigned a prevalence value based on infection in neighbouring households only.

### Weighted local prevalence analysis

This method calculates parasite prevalence amongst all neighbours within 1 km of the index house, weighting the prevalence estimate according to the inverse of the distance of the neighbouring house to the index house [20]. While a form of spatial smoothing, an important distinction between weighted local prevalence and kernel smoothing is that individuals in the index household are not included in the weighted prevalence estimate. As for kernel prevalence estimates, the weighted local prevalence for each household ranged from 0 (least exposed households) to 1 (most exposed households). As this method does not include infection status of individuals in the index household in the calculation of prevalence, no further action was required for those households with data from only the second year.

### Statistical analysis

To compare the ability of different cluster detection methods to predict infection in the second year, mixed effect logistic regression models was used. The outcome of interest was infection status by nPCR (0/1) in the second year. The risk factors explored were nPCR, AMA-1, MSP-1_19_ and AMA-1 and/or MSP-1_19_ (hereon termed combined seroprevalence) cluster score in the first year (generated via each of the three cluster detection methods). Simple summary contingency tables, graphs and scatter plots with Lowess curves were used to explore the relationship with potential risk factors and their associations with age. To explore the possibility of a non-linear relationship, risk factors were categorized into quartiles and a likelihood ratio test was used to assess which model (linear or categorical) was better. A household level random effect was included in the models to take account of correlation between individuals within the same household. All models were controlled for potential confounding by age, which due to an obvious non-linear relationship with infection was categorized before analysis into –zero to four years, five to nine years, ten to 15 years, 16-25 year, 26-35 years and over 36 years (Table 1).

**Table 1:** **Age**-**dependency of malaria in the baseline and follow**-**up surveys**

**Outcome**	**Age (years)**	**Total in each group**	**% positive**	**OR 95% CI**	**Wald test P value**
Infection by PCR (baseline survey)*	0-4	788 [27.5]		1	<0.001
	5-9	622 [47.9]		2.80 [2.17-3.62]	<0.001
	10-15	413 [50.1]		3.26 [2.44-4.35]	0.005
	16-25	409 [33.7]		1.52 [1.13-2.04]	0.721
	26-35	328 [26.5]		0.94 [0.68-1.30]	0.007
	36+	496 [20.6]		0.66 [0.49-0.89]	
**Infection by PCR (follow-up survey)**	0-4	824 [42.4]		1	<0.001
	5-9	644 [68.8]		4.77 [3.52-6.47]	<0.001
	10-15	359 [70.2]		5.58 [3.84-8.10]	<0.001
	16-25	445 [52.8]		1.96 [1.41-2.73]	0.661
	26-35	337 [44.8]		1.08 [0.75-1.56]	0.393
	36+	637 [39.7]		0.87 [0.63-1.20]	
**AMA** -**1 seropositivity (baseline survey)**	0-4	688 [21.7]		1	<0.001
	5-9	517 [53.0]		5.13 [3.84-6.86]	<0.001
	10-15	321 [64.2]		8.87 [6.29-12.50]	<0.001
	16-25	354 [60.2]		7.60 [5.47-10.56]	<0.001
	26-35	294 [51.0]		4.60 [3.29-6.42]	<0.001
	36+	416 [50.5]		4.39 [3.24-5.96]	
**MSP**-**1 **_**19 **_**seropositivity (baseline survey)**	0-4	698 [14.5]		1	0.111
	5-9	568 [16.9]		1.31 [0.94-1.84]	<0.001
	10-15	346 [30.6]		3.21 [2.24-4.59]	<0.001
	16-25	361 [34.9]		3.90 [2.75-5.51]	<0.001
	26-35	291 [38.5]		4.90 [3.39-7.07]	<0.001
	36+	447 [40.3]		5.10 [3.66-7.10]	

*age was missing for one individual.

To establish the effect of radius size on results obtained with the kernel and weighted local prevalence methods, models using different radii were built. In addition to the initial 1 km radius, radii of 500 m, 100 m and 0 m (i e, household) were explored. Models assuming individual level infection and serological status were also compared. Similarly, for the SaTScan analysis, maximum population sizes of 20 and 10% were explored. To compare the predictive performance of using different methods and radii, the area under the receiver operating curve (AUC) was calculated for each model. AUC values were compared using DeLong’s test for paired ROC curves [30]. Statistical analysis was performed using STATA (version 12, College Station, TX, USA) and R (version 3.0.1) [31].

## Results

### Study subjects

In 2010, 668 households from randomly selected sub-villages participated in the first year survey, comprising a total of 3,801 individuals, 3,057 (80.4%) of whom were seen, consented to participate and provided a blood specimen. Approximately half of the participants (n = 1,612, 52.7%) were male. The median age of the study population was 13 years (IQR = 5-30 years; range 1-99 years). The overall prevalence of *P. falciparum* by nPCR was 34.3%. In the second year survey, 697 households participated in the survey with 3,246 (85.4%) of eligible individuals providing a blood specimen, 51.6% of whom were male. Distribution of age was similar to that of the first year survey. *P. falciparum* prevalence by nPCR was significantly higher at 51.9% than during the baseline survey (OR 1.95; 95% CI, 1.76-2.17; p <0.001).

### Association of age and other individual factors with PCR positivity and seropositivity

Individuals aged 10 to 15 years had the highest nPCR prevalence of *P. falciparum* at baseline and at follow-up (Table 1). Seropositivity to AMA-1 similarly peaked in the age group ten to 15 years. This age group had more than eight times the odds of being seropositive to AMA-1 compared to individuals aged zero to four years (OR 8.87, 95% CI 6.29-12.5; P < 0.001). Seropositivity to MSP-1_19_ showed a different relationship with age, displaying a steady increase with age, with those aged >36 years having roughly five times the odds of being seropositive compared to those aged zero to four years (OR 5.10 95%, CI 3.66-7.10) (Table 1).

### Prediction of infection in the second year survey

#### nPCR prevalence in the baseline survey

Fifty-seven per cent of individuals who were nPCR positive in the first year were also nPCR positive in the second year whilst 47% who were negative in the first year were also negative in the second year (χ^2^ = 27.2; P <0.001). Guided by AUC values, clustering estimated using kernel analysis appeared to predict infection by nPCR in the second year more accurately than the weighted local prevalence method (p = 0.016) (Table 2). While clustering estimated by SaTScan gave a higher AUC value than clustering by the weighted local prevalence method, there was no evidence for a difference in AUC (p = 0.12).

**Table 2:** **Odds of testing positive for *P. falciparum *** **infection during the follow**-**up survey**: **results from three geospatial models defined by baseline infection**, **anti**-**AMA**-**1 antibody prevalence**, **and anti MSP**-**1**_**19 **_**antibody prevalence adjusted for age**

**Risk factor**	**Number tested**	**Malaria in second year n. %**	**OR 95% CI**	**Wald test P-value**	**Area under the ROC curve**
PCR individual infection in baseline survey*					
Neg	1,763	827 [46.9]	1	<0.001	0.560
Pos	905	521 [57.6]	1.58 [1.31-1.83]		
**PCR prevalence**					
*Satscan exposure category*	792	319 [40.3]	1	0.181	0.620
coldspot	1,728	864 [50.0]	1.35 [0.87-2.09]	<0.001	0.628
neither	726	500 [68.9]	4.54 [2.68-7.72]	0.966	0.597
hotspot	804	390 [48.5]	1	0.013	
*Kernel exposure quartiles*	819	387 [47.2]	0.99 [0.60-1.64]	<0.001	
<14.9	818	331 [40.5]	0.53 [0.32-0.88]	0.165	
15-21.3	805	575 [71.4]	3.45 [2.06-5.75]	0.042	
21.4-27.1	816	420 [51.5]	1	0.003	
>27.1	794	344 [43.3]	0.69 [0.41-1.16]		
*Weighted exposure quartiles*	807	372 [46.1]	0.58 [0.35-0.98]		
<18.9	799	520 [65.1]	2.21 [1.31-3.73]		
19-23.2					
23.3-26.5					
>26.5					
**AMA**-**1 individual prevalence**					
No	1,262	594 [47.1]	1	<0.001	0.554
Yes	1,071	593 [55.4]	1.45 [1.21-1.72]		
**AMA**-**1 prevalence**					
*Satscan exposure category*	904	310 [34.3]	1	<0.001	0.647
coldspot	1,092	554 [50.7]	2.65 [1.69-4.15]	<0.001	0.618
neither	1,250	819 [65.5]	5.84 [3.75-9.10]	0.002	0.609
hotspot	814	308 [37.8]	1	<0.001	
*Kernel exposure quartiles*	813	414 [50.9]	2.26 [1.35-3.79]	<0.001	
<27.9	812	425 [52.3]	2.62 [1.57-4.39]	0.154	
28-38.9	807	536 [66.4]	5.16 [3.06-8.69]	<0.001	
39-53.0	804	325 [40.4]	1	<0.001	
>53.0	809	357 [44.1]	1.45 [0.86-2.44]		
*Weighted exposure quartiles*	800	476 [59.5]	3.50 [2.07-5.91]		
<18.9	803	498 [62.0]	3.33 [1.97-5.62]		
19-23.9					
24 -26.9					
>26.9					
**MSP**-**1**_**19 **_**individual prevalence**					
No	1,730	924 [53.4]	1	0.196	0.541
Yes	681	341 [50.1]	0.88 [0.73-1.06]		
**MSP prevalence**					
*Satscan exposure category*	1,703	992 [58.2]	1	0.040	0.591
coldspot	967	493 [51.0]	0.64 [0.41-0.98]	<0.001	0.622
neither	576	198 [34.0]	0.21 [0.13-0.34]	0.773	0.625
hotspot	806	418 [51.9]	1	0.008	0.631
*Kernel exposure quartiles*	835	440 [52.7]	1.08 [0.65-1.78]	<0.001	
<12.9	808	538 [66.6]	2.02 [1.21-3.38]	0.715	
13-17.3	797	287 [36.0]	0.34 [0.20-0.55]	0.006	
17.4-25.4	805	415 [51.6]	1	<0.001	
>25.4	813	430 [52.9]	1.10 [0.66-1.81]		
*Weighted exposure quartiles*	802	533 [66.5]	2.08 [1.23-3.51]		
<16.5	796	278 [34.9]	0.35 [0.21-0.57]		
16.6-18.3					
18.4-22.7					
>22.7					
**MSP**-**1**_**19 **_**&/or AMA-1 individual prevalence**					
No	986	253 [25.6]	1	0.986	0.530
Yes	1,237	466 [37.7]	1.00 [0.78-1.29]		
**MSP**-**1**_**19 **_**&/or AMA**-**1 prevalence**					
*Satscan exposure category*	-	400 [48.4]	-	-	-
coldspot	-	357 [44.6]	-	-	0.604
neither	-	386 [47.0]	-	-	0.530
hotspot	827	540 [67.8]	1	0.082	
*Kernel exposure quartiles*	800	402 [49.3]	0.63 [0.37 - 1.07]	0.310	
<44.5	822	380 [47.4]	0.77 [0.46-1.28]	0.001	
44.6-51.4	797	372 [45.6]	2.44 [1.44-4.14]	0.507	
51.5-59.3	816	502 [64.0]	1	0.063	
>59.4	801		0.84 [0.49-1.42]	0.023	
*Weighted exposure quartiles*	815		0.60 [0.36-1.03]		
<16.5	784		1.86 [1.09-3.18]		
16.6-18.3					
18.4-22.7					
>22.7					

*Only individuals who were tested at both baseline and year 1.

Using SaTScan analysis to detect nPCR hotspots, one large cluster was identified with a radius of 2.88 km, covering 141 households and one small cluster was identified with a radius of 0.1 km covering five households (Figure 1A). SaTScan analysis showed that individuals who were residing in a nPCR hotspot cluster in the first year had four times the odds of testing positive for malaria by nPCR in the second year than those residing in nPCR coldspots (OR 4.54 95% CI 2.68-7.72). The kernel and weighted local prevalence analyses showed a more complex distribution of hotspots (Figure 1B and C). Both clearly show the central hotspot detected by SaTScan, but also show numerous other high transmission areas, more consistent with the micro-epidemiology of malaria. The kernel analysis also showed that individuals who were residing in the top quartile (areas with a high prevalence of infection by nPCR) had three times the odds of testing positive for malaria by nPCR in the second year compared to those living in the lowest quartile (OR 3.45, 95% CI 2.06-5.75).

#### Seropositivity to AMA-1 and MSP-1_19_ antibodies

Defining clusters of seroprevalence using AMA-1 and MSP-1_19_ antibodies separately improved prediction of nPCR positivity in the second year compared to using combined seroprevalence. SaTScan analysis revealed that individuals living in areas of high AMA-1 seroprevalence (hotspots) in the first year had five times the odds of being nPCR positive in the second year compared to those who lived in AMA-1 coldspots (OR 5.84 95% CI 3.75-9.10), adjusting for age (Table 2). SaTScan could not identify any significant clusters using combined seroprevalence.

**Figure 2:**
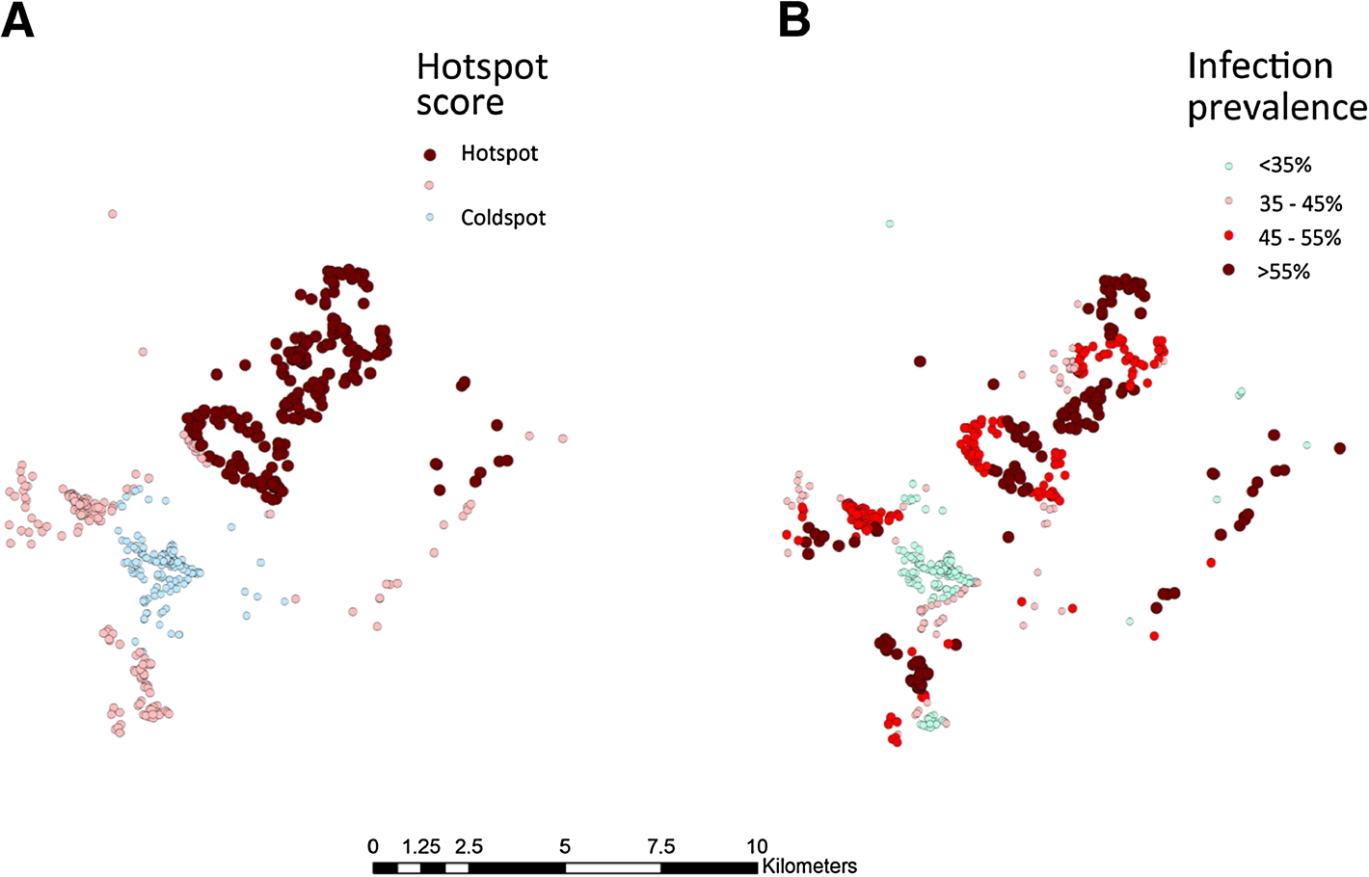
**Clustering of sero-positivity to AMA-1 in 2010 using SaTScan and kernel Method.** Clustering of sero-positivity to AMA-1 in 2010 using **(A)** SaTScan and **(B)** kernel with a 1 km radius.

When clusters were identified by kernel analysis, those individuals living in households with the highest quartile of AMA-1 seroprevalence (hotspots) had a more than five times the odds of being nPCR positive in the second year than those in the lowest quintile (OR 5.16 95% CI 3.06-8.69), adjusting for age (Table 2). Using weighted local prevalence scores to distinguish clusters showed a similar pattern, those residing in the households in the top quartile of AMA-1 seroprevalence (hotspots) had more than three times the odds of being nPCR positive than those residing in lowest quartile (OR 3.33 95% CI 1.97-5.62) (Table 2). Likewise the kernel analyses showed a more complex distribution of AMA-1 hotspots than SaTScan analysis (Figure 2). A comparison of the predictive ability of different clustering methods showed that both SaTScan and kernel analysis yielded higher AUC values than the weighted prevalence method, however, only the SaTScan method produced a significantly different result (p = 0.002 and p = 0.27 respectively).

Antibody responses to MSP-1_19_ showed a less clear association with infection in the second year, with individual age-adjusted seroprevalence at baseline showing no relationship with infection status in the second year. SaTScan analysis suggested that individuals living in MSP-1_19_ hotspots were at lower risk of infection in the second year. Both kernel and distance weighted prevalence analysis also suggested individuals living in areas of highest MSP-1 seroprevalence were at lower risk of infection, however those living in areas of intermediate seroprevalence (third quartile) were at higher risk of subsequent infection.

Individual seropositivity at baseline to the combined seroprevalence of AMA-1 and/ or MSP-1_19_ antibodies showed no relationship with infection in the second year. Similar to results using just AMA-1, kernel analysis of combined seroprevalence showed that those individuals living in the highest quartile had more than two times the odds of being nPCR positive in the second year than those residing in the lowest quintile (OR 2.44 95% CI 1.44-4.14). While a similar relationship was seen if hotspots were determined by weighted local prevalence, overall predictive ability using this method was worse than when using kernels with an AUC value of 0.530 (Table 2). SaTScan was not able to find any hotspots or coldspots using combined seroprevalence.

#### Sensitivity analysis of kernel and SaTScan methods for determining the best radius to predict malaria in the second year of follow-up

Based on AUC values, the weighted local prevalence method to identify clusters was generally less predictive of infection in the second year than the SaTScan and kernel methods. Sensitivity analyses of these two methods were therefore conducted to determine the radius size that best predicted infection in the second year. For the kernel method, using larger radii to identify clusters of nPCR tended to produced similar AUC values than smaller radii (Table 3). Using larger radii of 500 m and 1 km to identify clusters of AMA-1 seroprevalence, MSP-1_19_ or the antigens combined, generally produced higher AUC values. Similar sensitivity analyses were done for SaTScan, whereby the maximum population size allowable was set to 20 and 10%. As for the kernel analysis, there was a general trend to suggest that a larger maximum population size of 50%, which allows for larger geographic clusters, was more predictive of subsequent infection than smaller maximum population sizes (Table 3).

**Table 3:** Sensitivity analysis of kernel and SaTScan analysis of PCR and serology prevalence for prediction of infection in the second year

	**KERNEL**				**SaTScan**		
	**Radius**				**Window population size**		
**Exposure category**	**<1 m (Household)**	**100 m**	**500 m**	**1**,**000 m**	**10%**	**20%**	**50%**
***PCR prevalence quartiles***	0.612	0.622	0.611	0.628	0.593	0.616	0.620
*Area under ROC*							
*Proportion of total nPCR positive in the highest quartile in second year*_΅_	30.1%	30.2%	33.1%	34.2%	27.1%	29.7%	29.7%
*Proportion of the total study population included highest quartile*	23.4%	22.7%	25.2%	24.8%	20.3%	22.4%	22.4%
***AMA***-***1 prevalence quartiles***	0.583	0.587	0.619	0.618	0.602	0.615	0.647
*Area under ROC*							
*Proportion of total nPCR positive in the highest quartile in second year*_΅_	26.6%	29.0%	31.8%	31.9	9.7%	28.9%	48.3%
*Proportion of the total study population included highest quartile*	22.6%	24.9%	24.8	24.9	6.72%	22.6%	38.0%
***MSP***-**1**_**19 **_ ***prevalence quartiles***	0.559	0.533	0.602	0.622	0.595	0.612	0.591
*Area under ROC*							
*Proportion of total nPCR positive in the highest quartile I second year*	22.7%	22.8%	19.6%	17.1%	9.4%	11.8%	12.0%
*Proportion of the total study population included in the highest quartile*	24.9%	24.6%	24.5%	24.5%	13.7%	17.7%	17.8%
***MSP***-**1**_**19 **_**&/or AMA**-***1 prevalence quartiles***	0.575	0.580	0.585	0.604	-	-	-
*Area under ROC*							
*Proportion of total nPCR positive in the highest quartile in second year*	28.7%	30.8%	31.1%	32.6%			
*Proportion of the total study population included highest quartile*	24.6%	24.7%	24.9%	24.5%			

΅ Proportion of total nPCR positives in the second year that are found in the highest quartile.

## Discussion

It has been suggested that if malaria transmission hotspots can be identified, targeting interventions can have a improved impact on transmission [7]. A number of previous studies have explored the use of geospatial techniques to identify clusters of transmission markers such as infection or seropositivity to selected antigens [13,14,18,28,32,33]. These studies show that households with active and historic exposure tend to cluster together geographically. It is less clear however, whether these clusters predict future infection and if so, which geospatial techniques and transmission indicators should be used for their detection. Using two consecutive years’ data, this study shows that clusters of infection and seropositivity to AMA-1 are predictive of future infection and that kernel analysis and SaTScan are superior to the weighted local prevalence method of cluster detection.

Several authors have identified the existence of hotspots at single time points, using a variety of different measures of transmission [13,18,28]. Fewer studies have shown that hotspots are stable over time. Using data from multiple years in Kenya, Bejon *et al*. applied spatial scan statistics to identify infection hotspots that were predictive of future hotspots up to seven years later [14]. Another study done in a highland of Kenya by Ernst et al. identified stable spatial clusters of malaria cases by SaTScan statistics over a period of four years [33]. Again using spatial scan statistics, Bousema *et al*. showed that over the period of two years, clinical episodes of malaria cluster into hotspots [13]. This study is consistent with these findings, showing that hotspots of infection are predictive of future infection. The study also shows that being seropositive to AMA-1 or being in a hotspot of AMA-1 seroprevalence is predictive of future infection. As seropositivity to AMA-1 is indicative of recent exposure to *P. falciparum*, this finding adds further evidence that hotspots of transmission are stable over several years. The relatively low AUC values do, however, suggest the importance of other factors related to risk of infection that were not accounted for. In addition, the higher prevalence of infection seen in the second year, likely due to higher rainfall observed that year, led to some infections in non-hotspot households, which negatively impacts the AUC.

The relationship between hotspots of seropositivity to MSP-1_19_ and future infection was less clear. Clusters with high MSP-1 seroprevalence were found to be at lower risk of infection suggesting some protection at the neighbourhood level. However, whilst some studies have demonstrated a protective effect of antibodies to MSP-1_19,_[34-37] at the individual level, this was not observed in this study. The reasons for these observations and the differences in the patterns seen with AMA-1 require further investigation but they may relate to the differing immunogenicity and half-life of the antibody response to these two antigens [38].

In terms of methods to detect clusters, this study suggests that using spatial scan statistics or kernel analysis allows better characterization of hotspots than the weighted local prevalence method. This may be due to the fact that estimates of weighted local prevalence for each household are made using infection status of neighbours only. This likely leads to an inferior indication of hotspot location as individual or household level factors play an important role in risk of subsequent infection in that household. Sensitivity analyses, varying both the window size and maximum population size for kernel and SaTScan analysis respectively, suggests that generally hotspots form over larger (1-3 km) scales. While this likely varies by setting, similarly sized hotspots have been detected by previous studies in similar transmission settings [13,14,20]. In lower transmission settings, transmission appears to cluster over increasingly small scales. A recent study by Searle *et al*. in Zambia, where infection prevalence was estimated to be 23% by rapid diagnostic test (RDT), showed that active case detection within a 500-m radius could identify 76% of all RDT-positive individuals [39]. A study in Swaziland, where transmission is extremely low (PCR-derived parasite prevalence <1%), suggested that infections tend to cluster within households of passively detected cases [9].

This study has several potential operational implications for malaria control. Firstly, given the apparent stability of hotspots, targeting clusters of infection and seropositivity to AMA-1 (and/or antigens with similar properties) with complete cure treatment and vector control could have a dramatic impact on transmission [7]. Secondly, kernel analysis and SaTScan appear to be optimal methods to detect hotspots. Currently, establishment of seropositivity to AMA-1 can only be done using assays that require samples to be processed in the laboratory. Equally, while RDTs exist for determining infection status, these miss a large fraction of infections, most of which are likely to be subpatent [40-42]. Previous work has shown that these subpatent infections tend to cluster in hotspots, making RDTs inappropriate methods to detect hotspots [43]. In order to target interventions at hotspots, therefore, the development of sensitive rapid diagnostics for infection and seropositivity to AMA-1 (or similar) is required. Alternatively, it may be possible to identify hotspots in the field by clustering of particular risk factors or passively detected cases. This is the focus of further research. In the meantime, in the setting of moderate malaria transmission around Lake Victoria, mass drug administration of entire villages may be required to interrupt transmission [43].

### Limitations

This study used indirect measures to define household malaria exposure. Using more direct measures, such as entomological inoculation rate (EIR) and other vector measures, may have led to different results. However, EIR can be challenging to measure in low-endemic settings. Thus, individual parasite prevalence was chosen as the measure of subsequent transmission for this study. In addition, indoor residual spraying (IRS) was applied between survey periods throughout the study area. While there is no supporting data, it is likely that households that did not receive IRS were randomly distributed and therefore unlikely to introduce bias into the results. Lastly, the study continued for only two years, thus stability of malaria hotspots could only be predicted for that time period. However, as stated, the fact that hotspots of AMA-1 seroprevalence were predictive of future infection suggests transmission hotspots are stable over a longer time frame.

## Conclusions

This study supports previous work showing that hotspots can be defined using geospatial methods and are stable over a period of at least one year. Hotspots can be detected either by using parasite prevalence or seroprevalence of AMA-1 antibodies. It was also found that spatial scan statistics and kernel analysis were better at characterizing hotspots of transmission than the weighted local prevalence method. Given the lack of highly sensitive rapid diagnostic tests for infection and AMA-1 seropositivity, routine detection of hotspots is challenging. Further work exploring simple methods to identify hotspots with existing tools is therefore required. Furthermore, while theorized, it has yet to be shown in the field that targeting interventions does indeed lead to greater reductions in transmission over an untargeted approach. Studies linking methods of hotspot detection with assessments of the subsequent impact of targeted interventions would be extremely valuable.

## Competing interests

The authors declare that they have no competing interests.

## Authors’ contribution

JFM was involved in the study design, supervised the implementation of the study and data collection, analysed data, drafted and revised the manuscript. HJWS was involved in data analysis, interpretation of the data, drafted and revised the manuscript. DC and RDG were involved in overall study design and supervision, interpretation of the data and revisions of the manuscript. TB, CJS and CD were involved in supervision of laboratory work, interpretation of the data and revision of the manuscript. BG, JMB and KG were involved in interpretation of the data and revisions of the manuscript. NG, SA and SH performed the real time PCR testing, serology testing and revised the manuscript. All authors have read and approved the final version of the manuscript.
